# SET protein up-regulated testosterone production in the cultured preantral follicles

**DOI:** 10.1186/1477-7827-11-9

**Published:** 2013-02-19

**Authors:** Boqun Xu, Lingling Gao, Yugui Cui, Li Gao, Xue Dai, Mei Li, Yuan Zhang, Xiang Ma, Feiyang Diao, Jiayin Liu

**Affiliations:** 1 The State Key Laboratory of Reproductive Medicine, Clinical Center of Reproductive Medicine, First Affiliated Hospital, Nanjing Medical University, Nanjing, 210029, China; 2 Department of Obstetrics and Gynecology, The Second Affiliated Hospital of Nanjing Medical University, Nanjing, 210011, China

**Keywords:** SET, Androgen production, Preantral follicles

## Abstract

**Background:**

We found previously that the expression of SET gene was up-regulated in polycystic ovaries. Evidences suggested that SET protein was essential for regulating both the promoter activity of CYP17A1 and the biological activity of P450c17. In this study, we explored whether SET regulated androgen production in preantral follicles.

**Methods:**

The mouse preantral follicles were cultured *in vitro*. Testosterone secretion and expression of steroidogenic enzymes were observed in the preantral follicles treated *in vitro* by SET overexpression and knockdown.

**Results:**

Testosterone levels in the media of the AdCMV-SET infected follicles significantly increased, and the CYP17A1 and HSD3B2 expression also significantly increased (*P* < 0.05). Testosterone levels in AdSiRNA-SET infected group decreased, and so did CYP17A1 and HSD3B2 expression (*P* < 0.05).

**Conclusions:**

SET played a positive role in regulating ovarian androgen biosynthesis by enhancing the transcription of steroidogenic enzymes CYP17A1 and HSD3B2, which maybe contribute to the hyperandrogenism in PCOS.

## Background

The ovarian follicle develops as a functional unit which comprises of theca cells, granulosal cells, and oocyte. The theca cells are the primary source to synthesize androgen in response to LH [1-3]. Besides, the synthesis of androgen in theca cells is also regulated by a variety of additional steroidogenic enzymes. It is well known that theca cells express cytochrome P450 cholesterol side-chain cleavage enzyme (P450scc), 3β-hydroxysteroid dehydrogenase (HSD3B2), and cytochrome P450 cytochrome P450 17α-hydroxylase (CYP17A1) which has both 17α-hydroxylase and C17, 20 lyase activities [4]. All of these are key enzymes for androgen synthesis. In addition, steroidogenic acute regulatory (StAR) protein which facilitates cholesterol entry into the mitochondria mediates the rate-limiting step in the synthesis of androgen [5]. The regulation of these enzymes plays a critical role in normal androgen production and plays a proposed role in the pathogenic hyperandrogenic conditions such as Polycystic Ovarian Syndrome (PCOS) [6,7].

The SET protein (patient “SE translocation”), also known as TAF-1β [8], I2PP2A [9] and INHAT [10], belongs to a family of multitasking proteins, which is involved in apoptosis, transcription, nucleosome assembly, and histone binding. *SET* is located in chromesome 9q34 and encodes a 39 KDa phosphoprotein. It was originally identified as a translocated gene in acute undifferentiated leukemia [11]. In addition, as a transcriptional regulating factor, SET not only exerts by binding to the transcriptional co-activators CBP/p300 [12], but also acts directly as a transcriptional factor of CYP17A1 [13]. SET-mediated promoter hypoacetylation is a prerequisite for co-activation of the estrogen-responsive pS2 gene by PRMT1 [14]. SET is widely expressed in various tissues, especially in steroidogenic cells within the central nervous system, adrenal glands, and gonads [13,15,16]. In rat ovaries, SET protein expressed in theca cells and oocytes [15]. In human ovaries we found that SET protein also expressed predominantly in theca cells and oocytes (unpublished data). Recent evidence suggested that SET was essential for regulating both the promoter activity of CYP17A1 and the biological activity of P450c17. In human NT2 neuronal precursor cells and MA-10 cells, SET binds to rat CYP17A1 promoter at −418/-399 and activates its basal transcription from a rat CYP17A1 luciferase reporter plasmid [13,15,17]. In addition, SET has been identified as a potent inhibitor of the protein phosphatase 2A (PP2A). Miller et al. showed that, in NCI-H295A cells, SET can foster 17, 20 lyase activity of P450c17 by inhibiting the dephosphorylation action of PP2A on P450c17 [18], because the phosphorylation of P450c17 on serine and threonine residues increases its17, 20 lyase activity through as-yet-unidentified mechanisms, which may involve in increasing its affinity for *b*5 and/or P450 oxidoreductase [19].

Although the function of SET protein in some steroidogenic cells such as adrenal glands cells has been partly studied, the role of SET in androgen biosynthesis of ovarian theca cells has not been explored previously. Our previous studies have also found that SET was one of the overexpressed genes in polycystic ovaries compared to normal ovaries using cDNA microarray technology [20]. All of these attracted us to explore the function of SET in the regulation of ovarian androgen biosynthesis. This study may also help us to understand the pathophysiological role of SET in hyperandrogenism of PCOS.

## Methods

### Construction of recombinant adenovirus

The recombinant adenovirus was constructed using the AdEasy system [21]. Briefly, the cDNA sequence of SET was cloned by reverse transcriptive polymerase chain reaction (RT-PCR), and then subcloned into pAdTrack-CMV. The AdCMV-SET was recombined with backbone pAdEasy-1 in BJ5183 bacteria. The recombined adenovirus AdCMV-SET was generated and amplified in 293A cells. The titer of the viral stock was determined by the tissue culture infectious dose (TCID50) method. At the same time, the adenovirus of AdCMV was generated using “empty” pAdTrack-CMV vector as the control.

For endogenous SET knockdown experiments, the recombinant adenoviruses AdsiRNA -SET delivering siRNA targeting SET and AdsiRNA-Scrambled (control) viruses were constructed as previously described, with some modifications [22]. The potential target sequences for RNA interference were scanned with the siRNA Target Finder and Design Tool available on the Ambion Inc. website. The target sequence was as follows, SET SiRNA: 5^′^-ATATAACAAACTCCGCCAA-3^′^ (sense), SET Scramble: 5^′^-GAGACGTCAACTCCAACAC-3^′^. The target sequence was inserted into plasmid pShuttle-H1. The fragment H1-SiRNA/SET was digested and inserted into the plasmid pAdTrack-CMV to construct a shuttle plasmid containing reporter gene of green fluorescent protein (GFP). AdSiRNA-SET was recombined with backbone pAdEasy-1 in BJ5183 bacteria. The adenovirus of AdSiRNA-NS was constructed at the same time. Adenovirus generation, amplification, and titers were performed according to the methods described above.

### Follicle isolation and culture

All animal procedures were approved by the Institutional Animal Care and Use Committee of Jiangsu Province and were performed in accordance with the Guide for the Care and Use of Laboratory Animals. Our follicle culture *in vitro* methods had been established previously [23,24]. Briefly, ovaries were dissected immediately from 20-day-old female mice (Animal Centre of Nanjing Medical University, Nanjing, China) and transferred to the dissection medium, consisted of L-15 Leibovitz GlutaMAX (Gibco-BRL, Cergy-Pontoise, France) supplemented with 10% FCS, 100 IU/ml penicillin, and 100 μg/ml streptomycin that was prewarmed and maintained at 37°C. Ovaries were cleaned of connective tissues, and individual preantral follicles of 180–200 μm in diameter were isolated by manual dissection using 28.5-gauge needles (Becton Dickinson, Erembodegem, Belgium). Follicles were then transferred to the culture medium consisting of α-minimal essential medium (α-MEM; Gibco-BRL), 10% FCS, 100 mIU/ml recombinant follicle stimulating hormone (FSH; Gonal-F; Serono, Switzerland), and ITS with 5 mg/ml insulin, 5 mg/ml transferrin, and 5 ng/ml selenium (Sigma, USA). Only round follicles with intact basement membranes and theca layers were selected and placed individually in 96-well plates with 100 μl culture medium in each well and cultured at 37°C in a humidified atmosphere containing 5% CO2. Each treatment, respectively, was added in culture medium, as described in the results section. Specific treatments were performed in replicate and each experiment was repeated at least three independent times with a different preparation (pool) of follicles.

### Follicle treatment with the recombinant adenoviruses

On the first day of follicle culture, the media was replaced with fresh media. Recombinant adenoviruses AdCMV-SET, AdCMV-GFP, AdSiRNA–SET, and AdSiRNA-scrambled were then separately added to culture media with a multiplicity of infection (MOI) of 100 and evenly distributed by gentle shaking. Virus expression was monitored by fluorescence microscopy showing green fluorescent protein (GFP). The infected follicles cultured for 24 h were harvested for qRT-PCR, with a total of 20 follicles in each group. At forty-eight hours after infection, the follicles were collected separately and stored at −80°C for later Western blot analysis, with a total of 40 follicles in each group. At the same time, the culture media of those infected for 48 h, were collected and stored at −80°C for steroid content assay, with a total of 20 follicles in each group. All experiments were repeated at least three times.

### RNA extraction and qRT-PCR

RNA extraction of follicles was performed using the RNeasy Plus Micro Kit (Qiagen Inc, Chatsworth, CA) and total RNA was reverse transcribed with PrimeScript Reverse Transcription reagent Kit (Perfect Real Time), as described by the manufacturer. Amplification reactions were conducted using SYBR Premix Ex Taq (Perfect Real Time) with an ABI PRISM 7300 system. PrimeScript RT reagent Kit (Perfect Real Time) and SYBR Premix Ex Taq (Perfect Real Time) were purchased from TaKaRa Biotechnology (TaKaRa [Dalian] Co. Ltd, Dalian, China). Gene-specific qRT-PCR primers were used as listed in Table 1. GAPDH was employed as an internal control to normalize the loading templates of cDNA. Melting curve analysis was performed to confirm the specificity of qRT-PCR products. Each sample was assayed in duplicate and the fold changed in expression of each interested gene was analyzed via the 2^-ΔΔCt^ method.

**Table 1:** Primers used for qRT-PCR

**Gene**	**GeneBank ACC**	**Primer**	**Sequence(5**^**′**^**-3**^**′**^**)**	**Size**
SET	NM_023871	Forward	CTTCAAAGTCCACCGAAATCAAATG	146 bp
(mouse)		Reverse	ACCTGCGTCAGAATGGTCAGTAAAC	
GAPDH	NM_008084	Forward	AGGTTGTCTCCTGCGACTTCA	187 bp
(mouse)		Reverse	GGGTGGTCCAGGGTTTCTTACT	
StAR	http://NM_011485.4	Forward	CCACCTGCATGGTGCTTCA	142 bp
(mouse)		Reverse	TTGGCGAACTCTATCTGGGTCTG	
CYP11A1	NM_019779	Forward	GGCACTTTGGAGTCAGTTTACAT	186 bp
(mouse)		Reverse	GTTTAGGACGATTCGGTCTTTCTT	
CYP17A1	http://NM_007809.3	Forward	TCTGGGCACTGCATCACG	124 bp
(mouse)		Reverse	GCTCCGAAGGGCAAATAACT	
HSD3B2	http://NM_153193.2	Forward	CTGCGATCCAGAAACCTTCC	224 bp
(mouse)		Reverse	TCTTCCTCTTGCACCAACAAC	

### Hormone assays

Testosterone level in the collected media was assessed using the radioimmunoassay (RIA) kits (Beifang Biotechnique Institute, Beijing, China) according to the manufacturer’s protocol. The RIA kits were sensitive (<0.02 ng/ml) and reproducible (total coefficient of variation [CV], <10%) with measurement range from 0.1 to 20 ng/ml.

### Western blot analysis

The concentrations of protein extracted from the cultured follicles were measured by the Bradford method. Equal amount of protein was loaded and separated by 12% SDS-PAGE and then electroblotted onto Polyvinylidene fluoride membrane (Bio-Rad, Hercules, CA). The membranes were incubated with antibodies of target protein and then with HRP-conjugated secondary antibodies (1:1000; Beijing ZhongShan Biotechnology, China) and examined by enhanced chemiluminescence (Amersham Biosciences, Uppsala, Sweden). At last the results were analyzed by Quantity One version 4.6.2 (Bio-Rad, USA). The first antibodies used were: polyclonal antibody to human SET protein at 1:1000 dilution from ABCAM (Cambridge Science Park, Cambridge, UK); polyclonal antibody to mouse SET protein at 1:1000 from Santa Cruz Biotechnology (Santa Cruz, CA, USA); polyclonal antibody to β-tublin (as working calibrator) at 1:2000 from ABCAM (Cambridge Science Park, Cambridge, UK).

### Statistical analysis

Data were expressed as mean ± SD from at least three independent experiments. One-way ANOVA was used for statistical comparison. Values were determined to be significant when *p* < 0.05.

## Results

### SET overexpression promoted androgen production in theca cells

**Figure 1:**
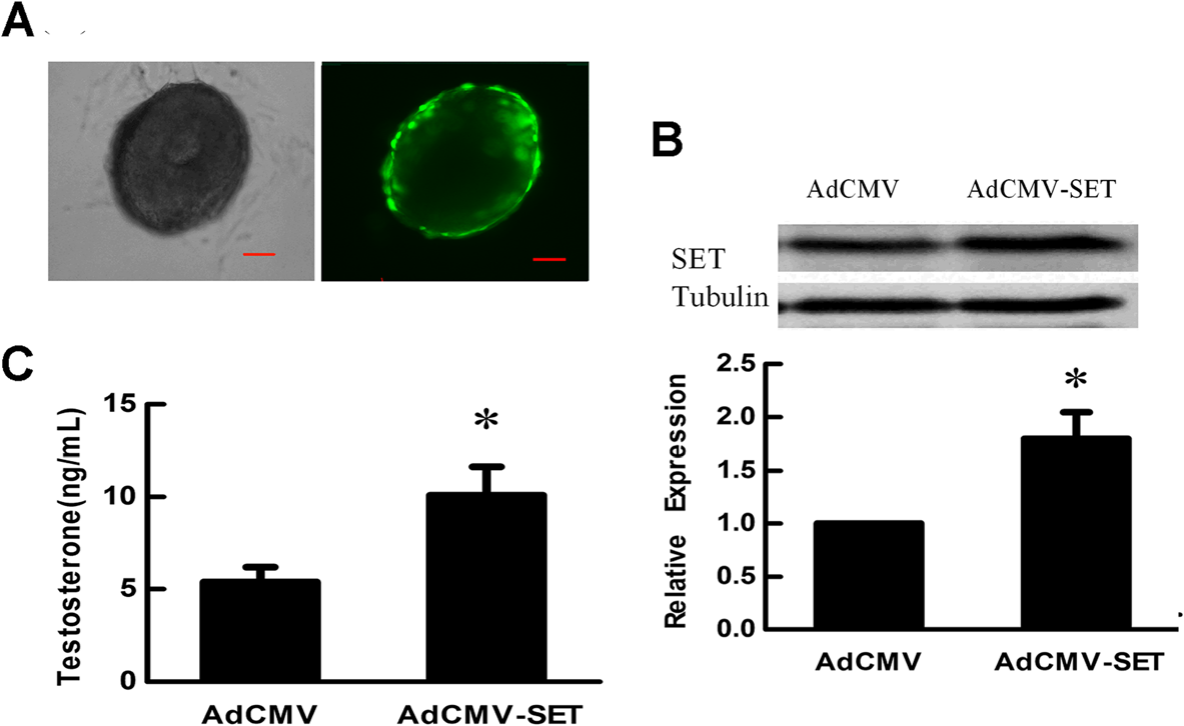
**Effect of SET overexpression on testosterone synthesis in theca cells.** (**A**) Infection of follicular theca cells with recombinant adenoviruses. Follicles were infected with recombinant adenovirus AdCMV-SET or AdCMV and cultured for 24 h. Over 90% theca cells were infected as judged by the expression of GFP under a fluorescence microscope. Left: light microscope; right: fluorescence microscope (Scale bar = 50 μm). (**B**) Western blot analysis of SET protein expression in follicles infected with recombinant AdCMV-SET adenovirus for 48 h. SET protein level in the follicles infected with AdCMV-SET were increased 1.8 times when compared with that in follicles infected with AdCMV. Tubulin was used as internal controls. (**C**) Effect of SET overexpression on testosterone synthesis in theca cells. Follicles were infected with AdCMV-SET or AdCMV for 48 h. Compared with that in the AdCMV infection group, testosterone production in the culture media of the AdCMV-SET infection follicles was significantly increased. Results are presented as Mean ± SD from at least 3 independent experiments. *, *p* < 0.05 compare with AdCMV.

The *in vitro* culture model of mouse preantral follicle was used to study the effect of SET on testosterone synthesis in theca cells. The recombinant adenovirus AdCMV-SET was constructed to up-regulate SET expression in theca cells of follicles. The report gene GFP was employed to indicate the efficiency of adenoviruses infection. At 24 h of post-infection, more than 90% of theca cells were infected as judged by the expression of GFP (Figure 1A). After 48 h, the expression of SET protein in follicles was analyzed by Western blot. SET protein in the follicles infected with AdCMV-SET was increased when compared with that in the follicles infected with AdCMV (*p* < 0.05, Figure 1B). To test whether SET regulated androgen production, the culture media of AdCMV-SET or AdCMV infected follicles were collected to evaluate testosterone levels. Compared with that in the AdCMV infected group, the testosterone level in the AdCMV-SET infected group was significantly increased (5.40 ± 0.80 ng/ml *vs* 10.09 ± 1.54 ng/ml, *p* < 0.05) (Figure 1C). These results indicated that SET promoted testosterone production in ovarian theca cells.

### SET knockdown suppressed androgen production in theca cells

**Figure 2:**
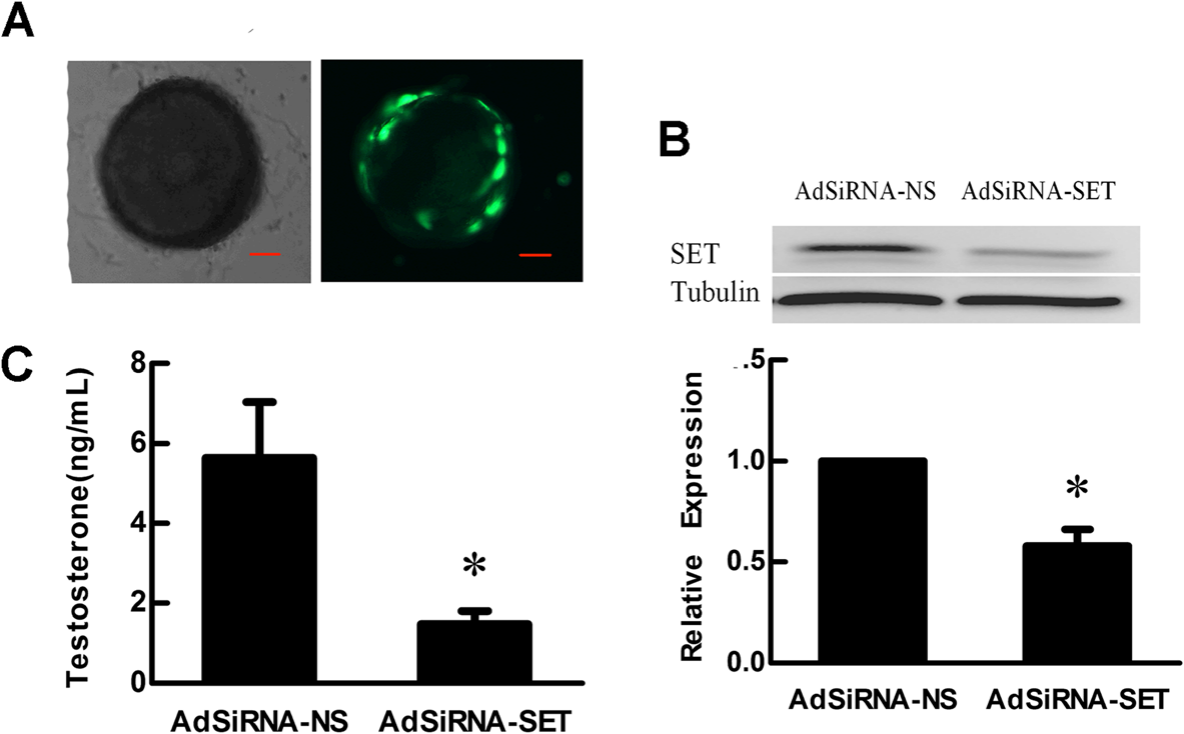
**Effect of SET knockdown on testosterone synthesis in theca cells.** (**A**) Infection of follicular theca cells with recombinant adenoviruses. Follicles were infected with recombinant adenovirus AdSiRNA-SET or AdSiRNA-NS and cultured for 24 h. The majority of theca cells were infected as judged by the expression of GFP under a fluorescence microscope. Left: light microscope; right: fluorescence microscope (Scale bar = 50 μm). (**B**) Western blot analysis of SET protein expression in follicles infected with recombinant adenovirus for 48 h. The levels of SET protein in follicles infected with AdSiRNA-SET were decreased about 43% compared with follicles infected with AdSiRNA-NS. (**C**) Effect of SET knockdown on theca cell testosterone synthesis. Compared with the follicles infected with AdSiRNA-NS, the amount of testosterone in the culture media of the AdSiRNA-SET infection group was significantly degraded. Results are presented as Mean ± SD from at least 3 independent experiments. Sample sizes were 20 follicles per treatment, per experiment. *, *p* < 0.05 compare with AdSiRNA-NS infected group.

To further confirm the effect of SET in modulating androgen synthesis in theca cells, analogous experiments were performed using AdSiRNA-SET and AdSiRNA-NS control adenoviruses. After 24 h of infection, more than 90% of theca cells were observed to express GFP (Figure 2A). As anticipated, level of SET protein was degraded about 43% in the follicles treated with AdSiRNA-SET when compared to that in the AdSiRNA-NS treated follicles (*p* < 0.05, Figure 2B). When compared with that in control group, testosterone accumulation in the media of AdSiRNA-SET infection group was significantly decreased (5.64 ± 1.40 ng/ml *vs* 1.49 ± 0.32 ng/ml, *p* < 0.05) (Figure 2C). This functional study strongly demonstrated that SET protein had a positive role in modulating testosterone synthesis in ovarian theca cells.

### SET regulated the transcription of steroidogenic enzymes in theca cells

**Figure 3:**
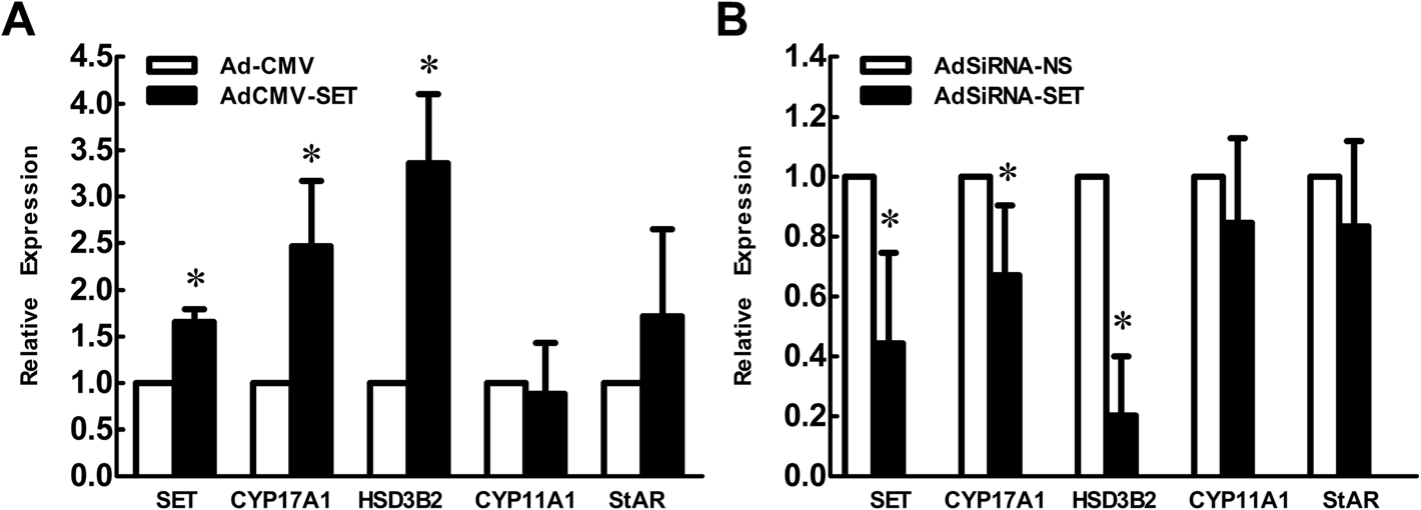
**Effect of SET in the regulation of steroidogenic enzyme expression in theca cells.** The cultured follicles were infected with recombinant adenoviruses for 24 h. The expression of SET, StAR, CYP11A1, CYP17A1 and HSD3B2 were analyzed by qRT-PCR. GAPDH were used as internal controls. (**A**) Compared to those receiving AdCMV, the SET mRNA of AdCMV-SET infected follicles was significantly increased and the expression of CYP17A1 and HSD3B2 were elevated. (**B**) The SET mRNA of AdSiRNA-SET infection group was significantly diminished compared with AdSiRNA-NS and the mRNA levels of CYP17A1 and HSD3B2 decreased significantly. Results were presented as Mean ± SD from at least 3 independent experiments. Sample sizes were 20 follicles per treatment, per experiment. *, *p* < 0.05 compared with the control adenoviruses infected group.

To determine whether SET regulated testosterone production by increasing the expression of steroidogenic enzymes in theca cells, the mRNA levels of SET and steroidogenic enzymes, StAR, CYP11A1, CYP17A1, and HSD3B2, in the follicles infected with AdCMV-SET or AdCMV, and AdSiRNA-SET or AdSiRNA-NS, were investigated by qRT-PCR. Compared to the AdCMV group, the mRNA levels of CYP17A1 and HSD3B2 in the AdCMV-SET infected group were significantly augmented following SET overexpression in theca cells (Figure 3A, *p* < 0.05). Additionally, down-regulation of SET with AdSiRNA-SET was accompanied by the significantly lowered mRNA levels of CYP17A1 and HSD3B2 (Figure 3B, *p* < 0.05). Expression of StAR and CYP11A1 in groups of both SET overexpression or knockdown did not change significantly (Figure 3, *p* > 0.05).

## Discussion

The biosynthesis of androgen in ovarian follicles is regulated by a series of steroidogenic enzymes, such as P450scc, StAR, HSD3B2, CYP17A1. These enzymes exert important functions in normal androgen production and play a proposed role in the pathogenic conditions such as hyperandrogenism in PCOS [6,7]. Previous studies showed that in PCOS theca cells the expression and activity of the P450c17 and 3β-HSD is elevated, and the CYP17A1 promoter activity is also increased [6,25-29], which partly participated in hyperandrogenism of PCOS. In the past years, much attention has been focused on the regulation of androgen biosynthesis, while the mechanisms that control the expression of these steroidogenic enzymes remain not precisely elucidated. In this work, we explored the role of SET protein in androgen biosynthesis and modulation of steroidogenic enzymes.

Previous studies have shown that SET was widely expressed in various tissues, and especially in steroidogenic cells within the central nervous system, adrenal gland, and gonads. To understand the function of SET in human ovaries, SET expression patterns had been examined by immunohistochemistry, which showed that SET was expressed predominantly in theca cells and oocytes of the human ovarian follicles (unpublished data). This was in agreement with previous studies performed by Zhang et al. [15] showing that SET was expressed in theca cells and oocytes of rats. Since the expressions of a variety of steroidogenic enzymes are cell specific, the primary function of theca cells is to produce androgen. The expression of SET in theca cells indicates that SET may be involved in androgen biosynthesis.

The *in vitro* culture model of mouse preantral follicles mimicked the native ovary by maintaining similar structural integrity. Hormones and paracrine factors produced by granulosal and/or theca cells can regulate the physiological function of cells located on the opposite side of the basement membrane. The gene-manipulation procedure was previously confirmed to effectively deliver ectopical gene expression in follicular theca cells [23]. In the present study, we developed the recombinant adenoviruses AdCMV-SET and AdSiRNA-SET to overexpress or knockdown SET gene in follicles. Our data firstly showed that overexpression of SET in theca cells promoted testosterone secretion. In contrast, down-regulation of SET led to a marked reduction in testosterone production. These results demonstrated that SET played a positive role in modulating testosterone secretion in follicular theca cells. In future study we will further examine the change of other androgens such as pregnenolone, androstenedione by chromatogram.

In ovarian theca cells, androgen biosynthesis is mediated by steroidogenic enzymes. Our further studies indicated that when SET was overexpressed in theca cells, expressions of CYP17A1 and HSD3B2 were significantly increased. As expected, the mRNA levels of CYP17A1 and HSD3B2 significantly decreased after SET was silenced in theca cells. Previous studies have shown that SET, as a novel transcriptional regulator, binds to the −410/-402 region of the rat CYP17A1 gene along with the transcription factors COUP-TF II, NGF-IB, and SF-1, and activates transcription of the rat P450c17 gene in neuronal precursor cells and mouse Leydig MA-10 cells [13,15,17]. Bioinformatical analysis showed that the SET binding sequences TCTCCTCAA is also present in the −312/-320 region of mouse CYP17A1 gene and a similar sequence ACTCCTCAG is present in the −964/-959 region of the mouse 3βHSD2 gene. Our present study indicated that SET may elevate the mRNA levels of CYP17A1 and HSD3B2 by promoting their transcription. Taken together, SET played a positive role in regulating ovarian androgen biosynthesis by enhancing transcription of CYP17 and HSD3B2, which may participate in hyperandrogenism of PCOS.

## Conclusions

SET played a positive role in regulating ovarian testosterone biosynthesis by enhancing the transcription of steroidogenic enzymes CYP17A1 and HSD3B2, which maybe contribute to the hyperandrogenism in PCOS.

## Abbreviations

CYP17A1: Cytochrome P450 17α-hydroxylase; GFP: Green fluorescent protein; HSD3B2: 3β-hydroxysteroid dehydrogenase; MOI: Multiplicity of infection; P450scc: P450 cholesterol side-chain cleavage enzyme; PCOS: Polycystic ovarian syndrome; PP2A: Protein phosphatase 2A; RT-PCR: Reverse transcriptive polymerase chain reaction; StAR: Steroidogenic acute regulatory; TCID50: Tissue culture infectious dose.

## Competing interests

The authors declare that they have no competing interests.

## Author’ contributions

B-QX carried out the experimental work and statistics. L-LG participated in molecular work. Y-GC participated in principal and experimental design. LG is the Lab assistant. XD participated in molecular work. ML participated in molecular work. YZ participated in cell culture work. XM participated in experimental design. F-YD participated in experimental discussion. J-YL participated in principal and experimental design. All authors read and approved the final manuscript.
